# Pedestrian Detection and Tracking from Low-Resolution Unmanned Aerial Vehicle Thermal Imagery

**DOI:** 10.3390/s16040446

**Published:** 2016-03-26

**Authors:** Yalong Ma, Xinkai Wu, Guizhen Yu, Yongzheng Xu, Yunpeng Wang

**Affiliations:** 1 Beijing Key Laboratory for Cooperative Vehicle Infrastructure Systems and Safety Control, School of Transportation Science and Engineering, Beihang University, Beijing 100191, China; mayalong@buaa.edu.cn (Y.M.); xinkaiwu@buaa.edu.cn (X.W.); yongzhengxu@buaa.edu.cn (Y.X.); ypwang@buaa.edu.cn (Y.W.); 2 Jiangsu Province Collaborative Innovation Center of Modern Urban Traffic Technologies, SiPaiLou #2, Nanjing 210096, China

**Keywords:** pedestrian detection, pedestrian tracking, aerial thermal image, video registration, unmanned aerial vehicle

## Abstract

Driven by the prominent thermal signature of humans and following the growing availability of unmanned aerial vehicles (UAVs), more and more research efforts have been focusing on the detection and tracking of pedestrians using thermal infrared images recorded from UAVs. However, pedestrian detection and tracking from the thermal images obtained from UAVs pose many challenges due to the low-resolution of imagery, platform motion, image instability and the relatively small size of the objects. This research tackles these challenges by proposing a pedestrian detection and tracking system. A two-stage blob-based approach is first developed for pedestrian detection. This approach first extracts pedestrian blobs using the regional gradient feature and geometric constraints filtering and then classifies the detected blobs by using a linear Support Vector Machine (SVM) with a hybrid descriptor, which sophisticatedly combines Histogram of Oriented Gradient (HOG) and Discrete Cosine Transform (DCT) features in order to achieve accurate detection. This research further proposes an approach for pedestrian tracking. This approach employs the feature tracker with the update of detected pedestrian location to track pedestrian objects from the registered videos and extracts the motion trajectory data. The proposed detection and tracking approaches have been evaluated by multiple different datasets, and the results illustrate the effectiveness of the proposed methods. This research is expected to significantly benefit many transportation applications, such as the multimodal traffic performance measure, pedestrian behavior study and pedestrian-vehicle crash analysis. Future work will focus on using fused thermal and visual images to further improve the detection efficiency and effectiveness.

## 1. Introduction

Pedestrian detection and tracking play an essential and significant role in diverse transportation applications, such as pedestrian-vehicle crash analysis, pedestrian facilities planning, the multimodal performance measure and pedestrian behavior study [1]. Many traditional methods for pedestrian detection and tracking use roadside surveillance cameras mounted on low-altitude light poles. For these methods, the coverage of the detection area is limited, and the installation is costly and time consuming. The unmanned aerial vehicles (UAVs) can be used as a high-altitude moving camera to cover a much wider area. Especially with the recent price drop of off-the-shelf UAV products, more and more researchers are exploring the potentials of using UAVs for pedestrian detection and tracking [2,3,4].

**Figure 1:**
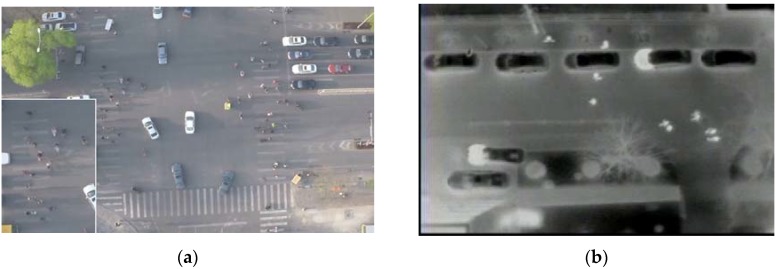
(**a**) Aerial optical image and (**b**) thermal image.

However, pedestrian detection from the images obtained from UAVs poses many challenges due to platform motion, image instability and the relatively small size of the objects. Depending on the flight altitude, camera orientation and illumination, the appearance of objects changes dramatically. This makes automatic object detection a challenging task. Using optical images, relatively larger objects, such as vehicles, can still be detected; but locating people presents an extremely difficult problem due to the small target size, shadow and low contrast of the target with the background or presence of clutter (see Figure 1a). To overcome this limitation, especially the potential optical camouflage, thermal imagery is employed, since the human thermal signature is somewhat more difficult to camouflage within the infrared spectrum [3]. However, even using thermal imagery, the detection of pedestrian trace is still a challenging problem due to the variability of human thermal signatures, which vary with meteorological conditions, environmental thermography and locales. As shown in Figure 1b, pedestrian objects in the thermal images appear as small hot blobs comprising only a few pixels and insufficient features, and non-pedestrian objects and backgrounds produce similar bright regions. All of these significantly downgrade the quality of the images and severely disturb accurate detection. In addition, the image instability caused by UAV jitter further increases the difficulty of detection.

This research aims to tackle the above challenges by proposing a pedestrian detection and tracking system using low-resolution thermal images recorded by the thermal infrared cameras installed in a UAV system. A two-stage approach, *i.e.*, Region of Interest (ROI) extraction and ROI classification, is first proposed for pedestrian detection. Given that the human body region is significantly brighter (or darker) than the background in the thermal images (see Figure 1b), a blob extraction method based on regional gradient features and geometric constraints is developed to extract bright or dark pedestrian candidate regions. The candidate regions are then fully examined by a linear Support Vector Machine (SVM) [5] classifier based on a weighted fusion of the Histogram of Oriented Gradient (HOG) [6] and Discrete Cosine Transform (DCT) descriptor for achieving accurate pedestrian detection. The experimental results illustrate the high accuracy of the proposed detection method.

With accurate pedestrian detection, this research then applies the pyramidal Lucas–Kanade method [7] to compute the local sparse optical flow, together with a secondary detection in the search region for correcting the drift, to track pedestrians. In order to eliminate the motion induced by UAVs, a semi-automatic airborne video registration method is applied before tracking. This semi-automatic registration method converts the spatiotemporal video into temporal information, thereby restoring the actual motion trajectory of pedestrians. The pedestrian velocity then can be derived from trajectory data.

The rest of the paper is organized as follows: Section 2 briefly reviews some work related to pedestrian detection and tracking in thermal images, followed by the methodological details of the proposed pedestrian detection and tracking system in Section 3. Section 4 presents a comprehensive evaluation of the proposed methods using diverse scenarios. Section 5 analyzes the pedestrian motion velocity. At the end, Section 6 concludes this paper with some remarks.

## 2. Related Work

### 2.1. Pedestrian Detection

Over the past two decades, considerable research efforts have been devoted to pedestrian detection in optical images [8,9], particularly using the HOG features first adopted by Dalal and Triggs. Concurrently, more and more research has focused on the effective detection of humans using thermal infrared images, mainly toward the applications of intelligent video surveillance [10,11,12,13,14,15,16] and Advanced Driver-Assistance System (ADAS) [17,18,19]. In [10], a two-stage template approach was proposed by employing a Contour Saliency Map (CSM) template with a background-subtraction method to obtain the location of a person, as well as examining the candidate regions using an AdaBoost classifier. In [11], the authors explored the human joint shape and appearance feature in infrared imagery. In their method, a layered representation was introduced to separate the foreground and background, and the shape and appearance cues were utilized for classification and localization, respectively. In [20], a DCT-based descriptor was used to construct the feature vector for person candidate regions, and a modified random naive Bayes classifier was applied to verify the person. The authors in [14] presented a background subtraction detection framework. The background image was extracted by various filtering and erasing of pedestrians, and pedestrians were segmented based on the size and histogram information. For a night-scene infrared video, the authors in [17] applied SVM classifiers using grayscale and binary features to recognize pedestrians from the candidate regions, and [18] proposed a tree-structured, two-stage Haar-like and HOG feature-based classifier to deal with the large variance in pedestrian size and appearance at night time for driver-assistance systems.

Very limited research has been conducted using images recorded by UAV systems for human detection, simply because the target size is too small in UAV images. The authors in [3] applied cascaded Haar classifiers with additional multivariate Gaussian shape matching for secondary confirmation to achieve people detection from UAV thermal images. The authors in [4] proposed a cascaded Haar feature-based body part detector by using the head, upper body and legs as the characteristic parts to generate individual body part classifiers. However, this method was evaluated by the images recorded by a stationary camera mounted on an elevated platform to replicate the UAV viewpoint. Considering that the human body parts in real UAV thermal images are not clear, the applications of this method are limited.

### 2.2. Pedestrian Tracking

Pedestrian tracking has been actively researched over the decades. Most of the existing methods are based on visual ground surveillance videos [12,21,22]. Considering the flexibility of aerial platforms and the large coverage of the scene from a top-down view, many researchers have begun to employ UAVs for object tracking. Our review here is only limited to approaches for pedestrian or object tracking with aerial platforms. The tracking of multiple objects from aerial platforms is a challenging topic because of the small size of objects, the low quality of images and the motion of the UAV. The authors in [23] combined background stabilization and layer representation to detect and track moving objects. They first applied a subtraction method to detect pedestrians, then employed the KLT (Kanade-Lucas-Tomasi) feature tracker and a post-linking process to track objects. The authors in [24] proposed a feature-based motion compensation algorithm, called image registration, to eliminate the motion of the aerial platform, and then adopted accumulative frame differencing to detect and track foreground objects. In [4], a particle filter-based approach was adopted to increase the identification and location accuracy of pedestrian objects. Furthermore, the authors in [25] explored a person tracking method for aerial thermal infrared videos using a combination of Harris corner detection and greedy correspondence matching. An OT-MACH (Optimal Trade-Off Maximum Average Correlation Height) filter is used in their method to determine whether a track is a person. However, when applying this method to aerial thermal images, the experiment results showed a poor performance due to the low quality of the images.

## 3. Pedestrian Detection and Tracking

### 3.1. Pedestrian Detection

**Figure 2:**
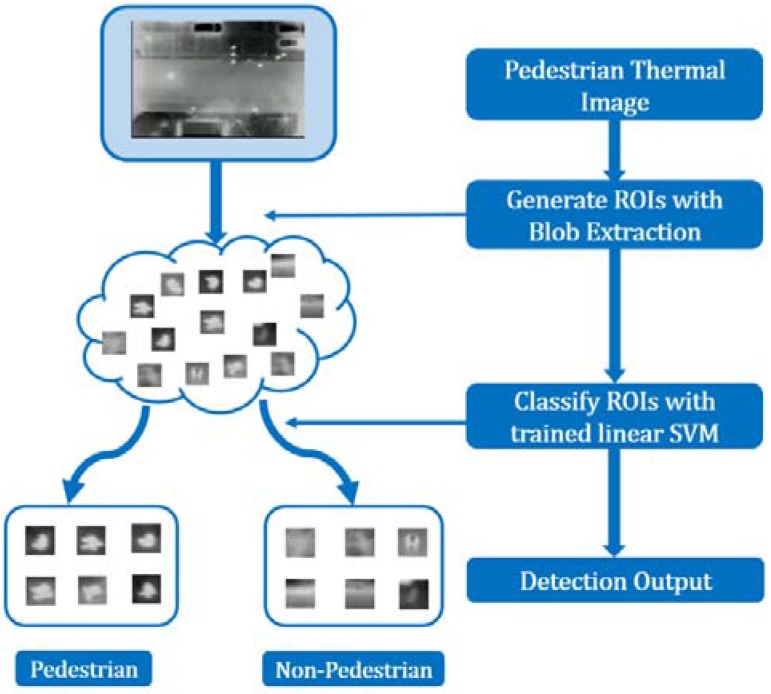
Pedestrian detection workflow.

A two-stage blob detection method is proposed in this research for pedestrian detection. The first stage is blob extraction. The proposed blob extraction approach applies the regional gradient feature and geometric constraint filtering to extract the precise pedestrian ROIs. Both bright and dark pedestrian regions can be detected. The second stage is blob classification. In this stage, a linear SVM classifier, which uses a weighted fusion of HOG and DCT descriptors as the feature vector, is utilized to verify the ROIs in order to achieve accurate detection. Figure 2 illustrates the basic workflow of the proposed detection framework.

#### 3.1.1. Blob Extraction

Essentially, pedestrian detection is a typical binary classification problem [3,9,17,18,20]. The general classification procedure includes three phases: ROI selection, feature descriptor extraction and region classification. The selection of ROI is critical for the overall detection, because any actual object missed in the ROI extraction stage will not be detected in the subsequent classification stage. A number of approaches have been proposed for pedestrian ROI detection, such as background subtraction [4,12,14,15], intensity contrast [17,18,20,26] and CSM representation [10,16]. Background subtraction heavily relies on the stationary camera and can only segment the moving foreground objects, thus limiting its expansion to aerial platforms. Intensity contrast methods take advantage of the fact that humans appear brighter than other objects in thermal imagery and apply several methods, such as the adaptive threshold segmentation [17,18] and Maximally-Stable Extremal Regions (MSER) method [13], to select ROIs. However, these methods easily lead to the fragmentation of the whole body or merge the human region with the bright background regions, therefore likely resulting in the failure of classification. The CSM shows the strong and significant gradient differences between foreground and background; but the same pedestrian object may have different saliency levels in different locations in the aerial thermal images (*i.e.*, non-uniform brightness), therefore creating difficulties in detection. To address the above problems and to take advantage of the fact that pedestrian objects appear blob shaped in aerial thermal top-views, a novel blob extraction approach, which uses the gray gradient feature of the region followed by geometric constraint filtering, is proposed to segment bright or black pedestrian regions.

**Figure 3:**
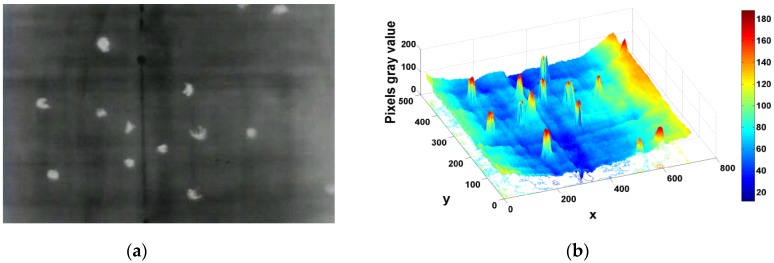
(**a**) Aerial thermal original image and (**b**) surface topographical picture.

**Figure 4:**
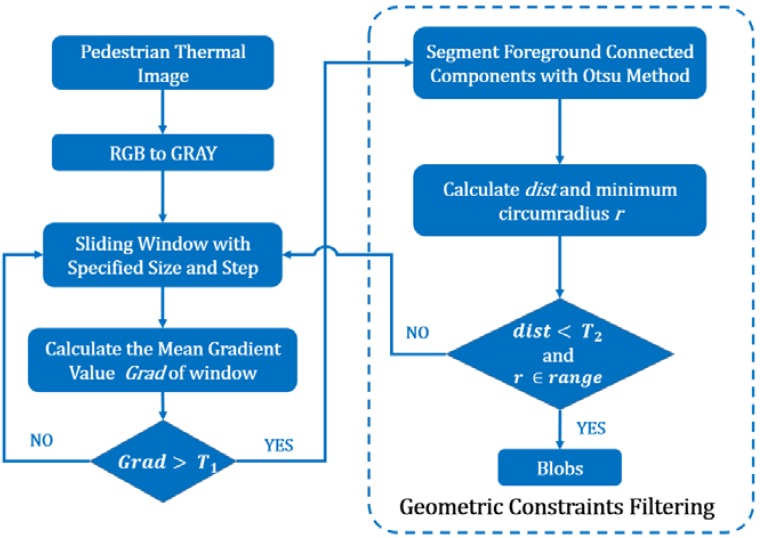
Blob extraction flow chart. $T_{1}$ is the predefined gradient threshold; $T_{2}$ is the predefined distance threshold; the range is the range of the minimum circumradius of a true pedestrian connected component; and *Grad* is the mean gradient value of local regions.

Generally, pedestrians appear brighter (or darker) than the surrounding background in thermal imagery. Figure 3a presents an example of pedestrians from a top-view aerial thermal image. As shown in the corresponding 3D surface topographical picture (see Figure 3b; the height indicates the gray value of pixels), the edge of the human region indicates an obvious gradient feature. Therefore, the basic idea of the proposed blob extraction method is to first separate human regions and non-human regions using edge gradient features. During this process, the sliding window method is applied to scan the image and to find ROIs. Figure 4 presents the overall flowchart of blob extraction. Some critical techniques are explained in the following section.

**Figure 5:**
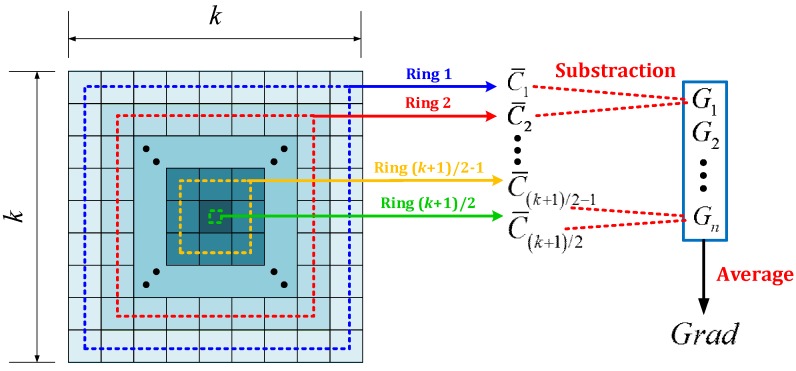
Illustration of *Grad* calculating process.

(1) Mean gradient value (*Grad*): First, each sliding window region is presented by a predetermined square matrix with k×k pixels (see Figure 5). To detect whether a local region is a pedestrian region, we simply need to check if the region is brighter (or darker) than the background. In other words, if a local region is a pedestrian region, the average gray value of inner rings should be larger (or lower) than the value of outer rings (see Figure 5). Note that due to the difficulty of detecting a pedestrian’s walking directions and walking states (e.g., arm swing and step type), we use the average gray value from all cells in a circle (*i.e.*, ring) in the matrix to represent the brightness level of the ring (see Figure 5). Then, the change of the brightness, *i.e.*, the gradient value $G_{i}$ between the average gray values from the *i*-th and (*i* + 1)-th ring, can be calculated by the following equation (see Figure 5):

(1) $$G_{i}={\bar{C}}_{i+1}-{\bar{C}}_{i}$$

where ${\bar{C}}_{i}$ represents the average gray value from all cells in the *i*-th ring. Given the size of the square matrix of k×k, the possible values of i are $i=\left[1,2,\ldots ,\frac{\left(k+1\right)}{2}-1\right]$. Note that when a pedestrian is colder than the environment, $G_{i}$ is calculated by the following equation:

(2) $$G_{i}={\bar{C}}_{i}-{\bar{C}}_{i+1}$$

With $G_{i}$, the average gradient value of a local region, *i.e.*, *Grad*, is calculated by:

(3) $$Grad=\frac{\sum_{i+1}^{n}G_{i}}{n}$$

where n represents the number of $G_{i}$ in a local region. For the case presented in Figure 5, n=(k+1)/2−1.

After calculating *Grad*, a predefined gradient threshold $T_{1}$ is used to detect if the region is a pedestrian ROI. In practical implementations, the integral image technology [27] is utilized to accelerate the computation of the average gray value of every ring. The fixed size sliding window is adopted to scan the image, because all pedestrian objects have almost the same scale in top-view thermal imagery, therefore consuming less time than using multi-scale scanning. Note here that the size of sliding window is relative to the flight height. Additionally, with the flight height increasing, the size of the sliding window should be decreasing. If the flight height is known, the size of the sliding window could be selected based on the following empirical equation:

k=[(110−h)/2]

where *k* (pixel) is the size of the sliding window and *h* (m) is the flight height. The notation of [x] indicates getting the largest integer less than or equal to x. Note that the flight height should be below 110 m.

(2) Geometric constraints filtering: Although nearly all pedestrian ROIs can be detected through the abovementioned approach, a large number of false ROIs that have the same gradient features as the true ROIs will also be extracted because of the use of the mean gray value. To filter the false ROIs, the Otsu threshold segmentation method [28] is employed to generate connected components for the pedestrian candidate in the binary image. The following two geometric constraints are used to refine the pedestrian candidate regions:

(1) Central location: According to the properties of a typical top-down view thermal image of a human, the distance between the centroid and center of the ROI should not be large. Therefore, the Euclidean distance between the centroid and center is calculated and then compared to a threshold ($T_{2}$) to filter ROIs (see Equation (4)):
  (4) $$\{\begin{array}{c} \mathrm{Pedestrian}\;\mathrm{ROI}:\;\mathrm{if}\;dist={∥P_{m}-P_{0}∥}_{2}\leq T_{2}\;\\ \mathrm{Non}-\mathrm{Pedestrian}\;\mathrm{ROI}:\;\mathrm{if}\;dist={∥P_{m}-P_{0}∥}_{2}>T_{2} \end{array}$$
  where $P_{m}$ and $P_{0}$ are the centroid and center of a square ROI, respectively.

This constraint eliminates most false pedestrian ROIs coming from the corners or edges of bright regions and the fragments of pedestrian blobs. Furthermore, this constraint ensures that the pedestrian is in the middle of the ROI, which is beneficial for subsequent classification.

(2) Minimum circumscribed circle: Because the scales of most of the pedestrian objects in top-view thermal images are similar, the pedestrian ROIs should not exceed a certain range. Therefore, by comparing the minimum circumradius *r* of a pedestrian connected component to a predefined range, we could eliminate too large or too small blobs and some rectangle regions. This method will further help filter the false ROIs.

**Figure 6:**
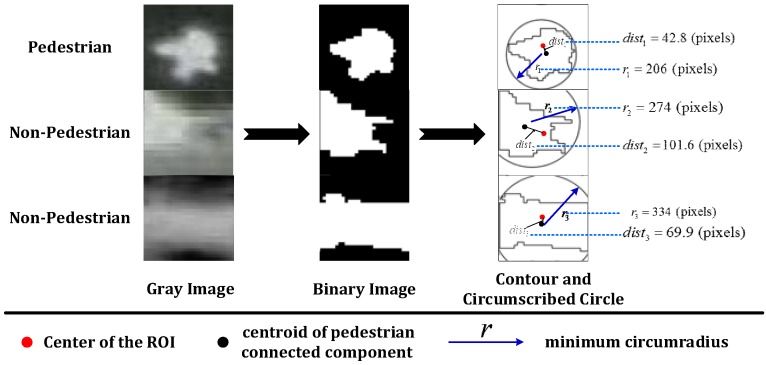
Illustration of geometric constraints. The first column is a gray image; the second column is the corresponding binary image; the third column is the image of the contour and minimum circumscribed circle: the red point is the center of the ROI; the black point is the centroid of the pedestrian connected component; dist is the Euclidean distance between the centroid and center; and r is the minimum circumradius represented by blue arrows; and the fourth column shows the examples of the values of *r* and *dist*.

Figure 6 illustrates two geometric constraints for both pedestrian and non-pedestrian ROIs. The example images are enlarged to 500 × 500 pixels for presenting the details. The first row represents the true pedestrian example, and other rows are non-pedestrian examples. From the figure, we can see that for non-pedestrian regions, either the distance between the centroid and center (*i.e.*, dist) is too large (see the case presented in the second row) or the circumscribed radius (*i.e.*, r) is too large (see the case presented in the third row). Therefore, by applying the above two filtering methods, most non-pedestrian regions can be eliminated, as shown in our testing presented in Section 4. In the practical extraction, for one blob, more than one window might be detected by the abovementioned procedures. We compute the average location for each group of windows. This reduces the subsequent classification times.

#### 3.1.2. Blob Classification

Blob extraction only detects pedestrian ROIs. To further classify the extracted blobs as pedestrians or non-pedestrians, a blob classification process is proposed here. A typical classification process consists of two steps: first, each blob is represented by a number of characteristics or denoted features; and second, some matching method is applied to compare the features of each blob with the features of the type of object that needs to be detected. In our research, due to the round shape of pedestrians in top-view thermal images, the pedestrian objects tend to be confused with other light sources, such as road lamps, vehicle engines, headlights and brushwood, leading a high false alarm rate. To overcome this difficulty, a unique descriptor, which combines both HOG and DCT features, is first used to describe extracted blobs, and then, a machine learning algorithm, SVM, is adopted to distinguish pedestrians from non-pedestrians objects. The following presents some details of the HOG and DCT descriptors and SVM.

(1) HOG descriptor: To achieve reliable and precise classification, the first step is to select appropriate features to form descriptors, which can be used to describe objects. Previous research has indicated that the HOG feature can be used to form a local region descriptor to effectively detect pedestrians in visual images. The HOG feature is based on the well-normalized local histograms of image gradient orientations in a dense grid. To extract HOG feature, the first step is to compute the gradient values and orientations of all pixel units in the image by applying the 1D centered point discrete derivative mask with the filter kernel [−1, 0, 1]. The second step is to create the cell histograms. In this step, the image is divided into cells, and the 1D histogram $H_{i}$ is generated by binning local gradients according to the orientation of each cell. The third step is to group cells together into larger, spatially-connected blocks $F_{i}$ and to normalize blocks in order to alleviate the influence of illumination and shadow. Since each block consists of a group of cells, a cell can be involved in several block normalizations for the overlapping block. Finally, the feature vector $V_{HOG}$ is obtained by concatenating the histograms of all blocks.

**Figure 7:**
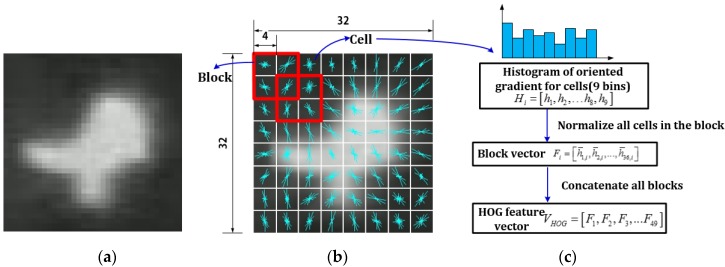
Illustration of HOG descriptor extraction. (**a**) Original image; (**b**) visualization of gradient orientations of all cells (white grids are cells, and red bounding boxes are block regions); and (**c**) generating the feature vector.

The HOG feature essentially describes the local intensity of gradients and edge directions. Since the shapes or edges of the pedestrian and non-pedestrian objects are obviously different, the HOG feature is applied to characterize the difference. In our experiments, the UAV flight altitude ranges from 40 m to 60 m, and the corresponding object size approximately ranges from 35 × 35 pixels to 25 × 25 pixels. Hence, we normalize the object size by scaling the examples to 32 × 32 pixels. We then use 8 × 8 blocks with a four-pixel stride step and nine histogram bins (each bin is corresponding to 20° of orientation) to calculate the feature vector. Note for an image with 32 × 32 pixels, a total of 49 blocks can be identified (note, 49 is calculated by ${\left(\frac{\left(32-8\right)}{4}+1\right)}^{2}=49)$. Therefore, the final HOG feature descriptor can be described by a vector $V_{HOG}$:

(5) $$V_{HOG}=\left[F_{1},F_{2},\cdots ,F_{i},\cdots F_{49}\right]$$

where $V_{HOG}$ is the HOG feature descriptor and $F_{i}$ is the normalized block vector for the *i*-th block. Since each block contains four cells and each cell contains nine bins, $F_{i}=\left[{\bar{h}}_{1,i},{\bar{h}}_{2,i},\ldots ,{\bar{h}}_{j,i},\ldots ,{\bar{h}}_{36,i}\right]$, where ${\bar{h}}_{j,i}$ is the *j*-th normalized value in the *i*-th block. Figure 7 illustrates the process of extracting the HOG descriptor.

(2) DCT descriptor: This research further explores the potential of using the DCT feature as the descriptor. DCT has been widely applied to solve problems in the digital signal processing and face recognition fields [29,30]. In addition, the DCT feature also showed good performance for low resolution person detection in thermal images [20].

DCT essentially transforms signals (the spatial information) into frequency representation. To derive a DCT descriptor, the first step is to calculate the DCT coefficients. Typically, for an M × N pixel image, the following equation can be used to calculate the DCT coefficient matrix *G*(*u, v*) at frequency component (u,v):

(6) $$G\left(u,v\right)=\frac{2}{\sqrt{MN}}\overset{M-1}{\underset{x=0}{\sum }}\overset{N-1}{\underset{y=0}{\sum }}C\left(u\right)C\left(v\right)f\left(x,y\right)\cos\left[\frac{\pi u\left(2x+1\right)}{2M}\right]\cos\left[\frac{\pi v\left(2y+1\right)}{2N}\right]$$

where *f*(*x*, *y*) is the intensity of the pixel in row x and column y in the input image. *C*(*u*), *C*(*v*) are defined by the following:

$$C\left(u\right),C\left(v\right)=\{\begin{array}{c} \frac{1}{2}\;u=0,v=0\\ 1\;otherwise \end{array}$$

**Figure 8:**
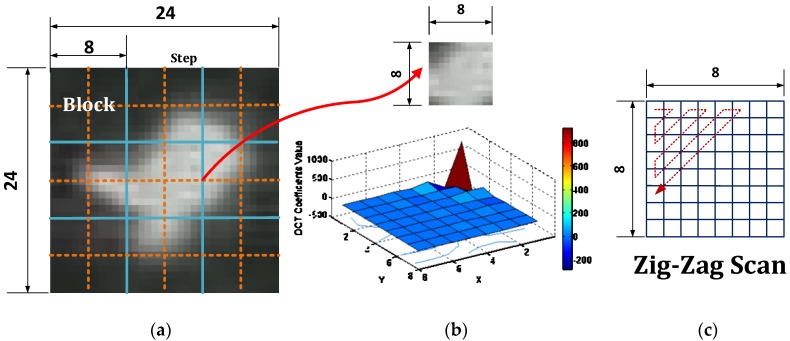
Illustration of the DCT descriptor extraction. (**a**) Original image division; (**b**) visualization of DCT coefficients value of block; (**c**) zigzag scan method.

Generally speaking, using the essential DCT coefficients as the descriptors can mitigate the variation of pedestrian appearance. For most of the pedestrian thermal images, signal energy usually lies at low frequencies, which correspond to large DCT coefficient magnitudes located at the upper left corner of the DCT coefficient matrix. In our experiments, the example image is first divided into blocks of 8 × 8 pixels size (see Figure 8a), then discrete cosine transform is applied to each block to obtain the corresponding DCT coefficient matrix (see Figure 8b), and at the end, the top-left DCT coefficients are extracted via zigzag scan (see Figure 8c). Note that only the first 21 coefficients in the matrix are selected as the feature vector of the block, because more coefficients do not necessarily imply better recognition results, and adding them may actually introduce more irrelevant information [29]. Then, for each block, a final feature vector, which concatenates the 21 low-frequency coefficients, is constructed. In addition, as mentioned before, most information of the original image is stored in a small number of coefficients. This is indicated by Figure 8b, in which the value of DCT coefficients at the matrix top-left is significantly larger than the others.

(3) SVM with a combination descriptor of HOG and DCT: Unfortunately, neither HOG nor DCT shows consistently good performance in our tests. To enhance the adaptability of feature descriptors, a weighted combination descriptor of HOG and DCT is proposed here. Because HOG can help capture the local gradients and edge direction information and DCT can help address the variation of object appearance, such as the halo effects and motion blur [20], combining both features is a legitimate idea. In our experiments, the HOG feature vector $V_{HOG}$ has been normalized by the L2-norm method. Therefore, the DCT feature vector $V_{DCT}$ needs to be normalized, as well, due to its large magnitude. In this research, the DCT feature vector $V_{DCT}$ is normalized using $v_{i}^{*}=v_{i}/\max\left(v_{1},v_{2},\cdots ,v_{n}\right)$. The final combination descriptor $V_{F}$ is constructed by the weighted combination of $V_{HOG}$ and $V_{DCT}$, *i.e.*, $V_{F}=\left[\alpha V_{HOG},\;\beta V_{DCT}\right]$. The values of α and β are hyper-parameters, which are determined by cross-validation, aiming to minimize the number of false classifications in training samples.

With a hybrid descriptor, SVM is then applied to classify pedestrian and non-pedestrian objects. SVM has been proven to be a highly effective two-category classification algorithm, which looks for an optimal hyper-plane to achieve maximum separation between the object classes. SVM demonstrates good performance on high dimensional pattern recognition problems. In our case, a linear SVM trained by example images from the blob extraction stage with and without pedestrians is used to classify the pedestrian candidate regions. Since the hybrid descriptor takes advantage of both HOG and DCT features of infrared thermal objects, our testing shows great improvements of the results (see Section 4).

Note that in our practice, we only employed a simple linear kernel without any parameter selection methods, such as cross-validation and grid search, due to the following two reasons: (1) the kernel type is difficult to determine; (2) the grid search or cross-validation would cost too much time in the training stage. Our experiment has shown that it takes days to search for the optimal parameters when using cross-validation and grid-search methods. Therefore, we believe that a simple linear kernel without any parameter selection methods works best for our detection framework.

### 3.2. Pedestrian Tracking

After detecting the pedestrian, a tracking approach is proposed here. Our purpose is to track multi-pedestrians simultaneously once they have been detected in the detection stage and to extract the trajectory data for the subsequent motion analysis. However, due to the irregular motion of the UAV in three axes of roll, pitch and yaw [31], the video pictures rotate and drift. This brings difficulties for pedestrian tracking and trajectory extraction. Therefore, to eliminate the motion of UAV surveillance videos, an image registration technology is applied first.

#### 3.2.1. Video Registration

Video registration aims to spatially align video frames in the same absolute coordinate system determined by a reference frame. It essentially converts the spatiotemporal information into temporal information. A typical feature-based alignment framework for video registration contains the following steps [24]:

(1) Detecting the stationary feature as the control points;
(2) Establishing correspondence between feature sets to obtain the coordinates of the same stationary point;
(3) Setting up the transformation model from the obtained point coordinate;
(4) Warping the current frame to the reference frame using the transformation model.

The registration algorithm is crucial to subsequent tracking, because errors induced by this step would impact the tracking accuracy. The proposed registration method uses the KLT feature [32], which has been proven to be a good feature for tracking. This whole process includes three steps: (1) KLT feature extraction; (2) KLT tracking; and (3) aligning using affine transformation.

KLT feature extraction is derived from the KLT tracking algorithm, which computes the displacement of the features between two small windows in consecutive frames. The image model can be described by the following equation:

(7) J(x)=I(x−d)+η(x)

where $x={\left[x,\;y\right]}^{T}$; *I*(**x**), *J*(**x**) represent the intensities of tracking windows centered at feature points in the current frame and the next frame, respectively; **d** represents the displacement vector; and η(x) is a noise term.

The goal for tracking is to determine **d** that minimizes the sum of squared intensity difference between *J*(**x**) and *I*(**x − d**) in the tracking window, as described by:

(8) $$\varepsilon =\iint {\left[I\left(x-d\right)-J\left(x\right)\right]}^{2}\omega dx$$

where ω is a weighting function. For the small displacement, the intensity function can be approximated by Taylor series as:

(9) I(x−d)=I(x)−g⋅d

where $g={\left[\partial I/\partial x,\partial I/\partial y\right]}^{T}$. Then, Equation (8) is differentiated with respect to **d**, and the result is set equal to zero. The final displacement equation can be expressed as follows:

(10) Gd=e

where *G* is a 2 × 2 coefficient matrix:

$$G=\iint {\mathrm{gg}}^{T}\omega dA$$

and *e* is the 2D vector:

e=∬(I−J)gωdA

Note that to obtain a reliable solution of Equation (10), eigenvalues of coefficient matrix *G* must be large [33]. The KLT feature then is defined as the center of window *W*, where the minimum eigenvalue of *G* is larger than a predefined threshold. These points usually are corners or have large intensity gradient variation.

**Figure 9:**
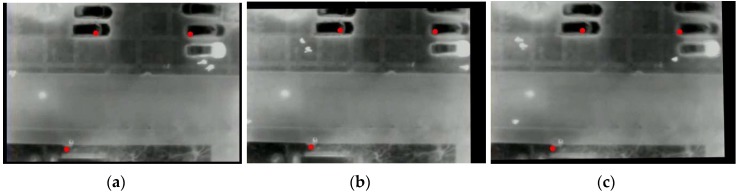
Illustration of video registration. (**a**) The reference frame (the first frame); (**b**,**c**) the 317th and 392nd frames mapping to (**a**), respectively. The red points are the KLT control points.

After the detection of the KLT feature in the first frame of the video, the control points for registration are selected manually to avoid finding false points on moving objects. Then, the KLT tracker is introduced to track the chosen feature points in the video sequence and to establish correspondence for computing the transformation model. We employ affine transformation to transform the current frame to the first frame, which needs four selected feature points. Mapping to the first frame avoids the error accumulation caused by registration between two consecutive frames. This KLT feature-based method can eliminate the small range motion of aerial videos. Figure 9 shows the registration example images.

#### 3.2.2. Pedestrian Tracking

After video registration, the KLT feature-based tracking method is applied for pedestrian tracking. Since most of the KLT features are located on the edge of the object, where the intensity difference exists, the KLT features are not accurate enough to describe the locations of pedestrians. To address this issue, the center of the detected pedestrian is determined as the beginning of tracking, and then, a pyramidal implementation of the Lucas–Kanade feature tracker is employed to accurately compute the displacement. Image pyramidal representation could handle relatively large displacement because the optical flow is calculated and propagates from the highest to lowest levels. In addition, due to the low quality of infrared imagery and motion blur caused by UAVs, the tracking points might drift away from the center during the tracking process. This is because the premise of optical flow methods assumes that the gray level of objects keeps consistent over time, but the thermal images cannot satisfy this assumption due to the high noise level and non-uniform brightness. The drift could significantly downgrade the tracking accuracy. To address this issue, a secondary detection stage in a local region followed by the feature tracking is used to correct the drift, as described in the following:

(1) Once one pedestrian object is detected in the current frame, input the center coordinate of the object into the feature tracker;
(2) Applying the pyramidal Lucas–Kanade feature tracker to estimate the displacement between consecutive frames, the estimated displacement then is used to predict the coordinate of the object in the next frame;
(3) Around the predicted coordinate, a search region is determined. In the search region, a secondary detection is conducted to accurately find the pedestrian localization.

Note that in the search region, more than one object may be detected. In this case, the minimum distance can be obtained by comparing all distances between the centers of detected objects and the predicted coordinate. If the minimum distance is less than half of the predefined blob size, the corresponding object center will be the corrected pedestrian location.

**Figure 10:**
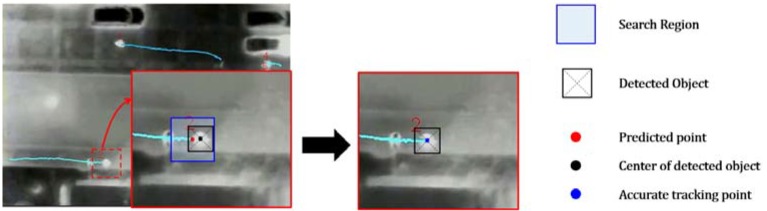
Illustration of the update of the secondary detection.

Then, the predicted point will be replaced by the detected pedestrian central point. Repeating the aforesaid process can keep the tracking continuous and accurate. Figure 10 illustrates the update process. Note that during the practice, a target may not be detected all of the time in the search region. If no pedestrians are detected in the search region, the tracking point will keep its origin, *i.e.*, the predicted point. This automatic feature tracker is crucial for providing us the coordinates of the corresponding point of the same object. In addition, to improve tracking efficiency, the detection of the whole image is only conducted every 15 frames for detecting the new pedestrian objects.

Overall, the proposed tracking method is similar to some tracking-by-detection methods, which are usually used in optical images and implemented using online learning. However, our detection scheme in the tracking approach utilizes the classifiers that have been trained during blob classification (see Section 3.1.2). This significantly saves time for real-time pedestrian tracking and improves the efficiency.

## 4. Experiments for Pedestrian Detection and Tracking

### 4.1. Evaluation for Pedestrian Detection

**Figure 11:**
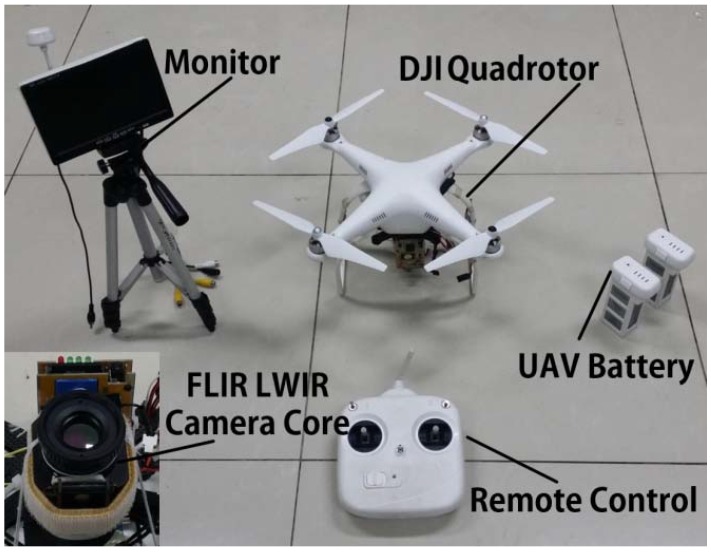
Basic components of the UAV platform.

To evaluate the proposed pedestrian detection approach, experiments were conducted using aerial infrared videos captured by a thermal long-wave infrared (LWIR) camera with a 720 × 480 resolution mounted on a quadrotor with a flight altitude of about 40 to 60 m above the ground. Figure 11 shows the basic components of UAV platform used in this research. Five different scenes with different backgrounds, flying altitudes, recording times and outside temperatures were used for evaluation (see Table 1). The detection statistical effect was evaluated using two measurements: (1) Precision=TP/(TP+FP), which is the percentage of correctly-detected pedestrian number over the total detected pedestrians; and (2) Recall=TP/(TP+FN), which is the percentage of correctly-detected pedestrian number over the total true pedestrians. Here, TP (*i.e.*, True Positive) is the number of correctly-detected pedestrians. FP (*i.e.*, False Positive) is the number of detected objects that are not pedestrians, and FN (*i.e.*, False Negatives) is the number of misdetected pedestrians. In our evaluation, a detected blob is regard as a True Positive (TP) when it fits the following condition:

$$\frac{A_{anno}\;\cap \;A_{det}}{\min\left\{A_{anno},\;A_{det}\;\right\}}\geq 0.7$$

where $A_{anno}$ denotes the area of the annotated ground-truth rectangle surrounding an actual pedestrian and $A_{det}$ is the area of the detected pedestrian blob.

**Figure 12:**
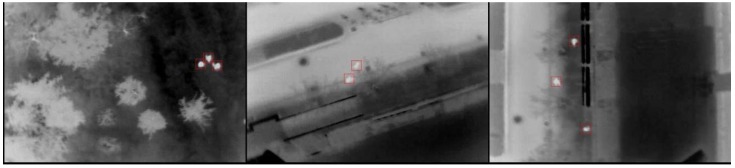
Pedestrian detection sample images for Scene 1 only using blob extraction.

For the simple background scene, such as grassland, a playground, *etc.*, there is high contrast between pedestrians and the background (see Figure 12). For this type of scene, selecting pedestrian sample images and training the classifier are not necessary, since the pedestrian targets can be easily detected by only using blob extraction. In our test, 176 typical frames were selected from these videos to form a test dataset named “Scene 1”. For this dataset, only blob extraction method was applied. The testing results are shown in Table 2. Note that the numbers in Table 2 indicate the sum of pedestrian numbers of all images in corresponding datasets. As shown in the table, the precision and recall rates are as high as 94.03% and 93.03%, respectively.

**Figure 13:**
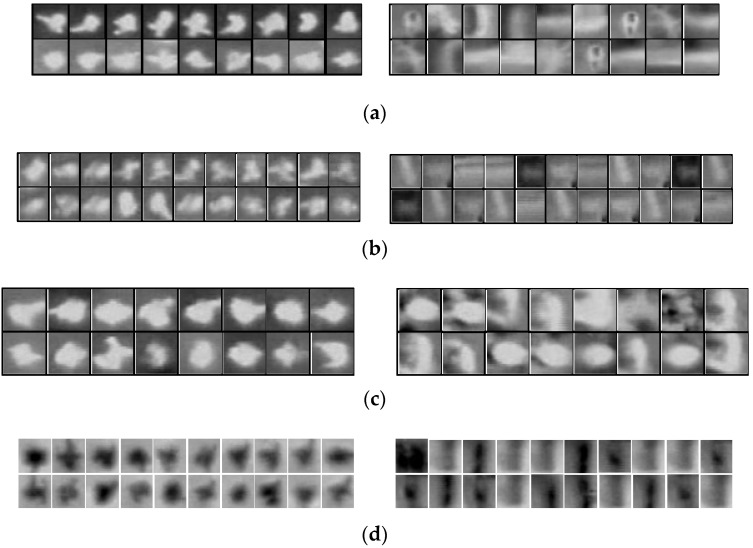
Pedestrian (left column) and non-pedestrian (right column) blob examples for: (**a**) dataset “Scene 2”; (**b**) “Scene 3”; (**c**) “Scene 4”; and (**d**) “Scene 5”.

For the other four scenes, the background is much more complex due to some non-pedestrian objects, such as lamps, vehicle engines, headlights and brushwood, which are similar to true instances. The supervised classification algorithm was employed to distinguish them. To evaluate the performance of the classifier, we set up four challenging datasets composed of typical images selected from four corresponding aerial videos, which may generate a number of false positives only using blob extraction. Table 1 shows the basic information of datasets. In our experiments, a part of the images from the dataset was chosen as the training images and the remainder as the test images. The pedestrian candidate regions generated by the blob extraction method (see Section 3.1.1) in training images were labeled manually for pedestrian and non-pedestrian blobs. Then, the blobs from training images were used to train the linear SVM classifier, and then, the blobs extracted from test images were used to examine the performance of the classifier. Table 3 provides the details of images for training and testing for four datasets. Some pedestrian and non-pedestrian example blobs from four datasets are also shown in Figure 13. As shown in the figure, the appearance of pedestrians varies across the datasets because of the different flight altitudes and temperatures. For each dataset, the performances of HOG, DCT and the proposed hybrid descriptors were compared. The Receiver Operating Characteristic (ROC) curves were drawn to reflect the performance of the combination of descriptors and linear SVM.

**Figure 14:**
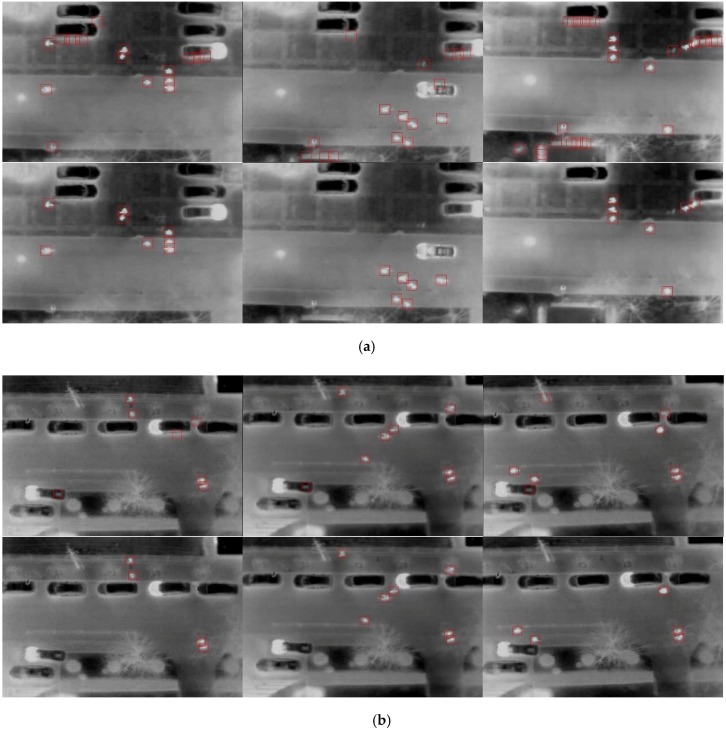

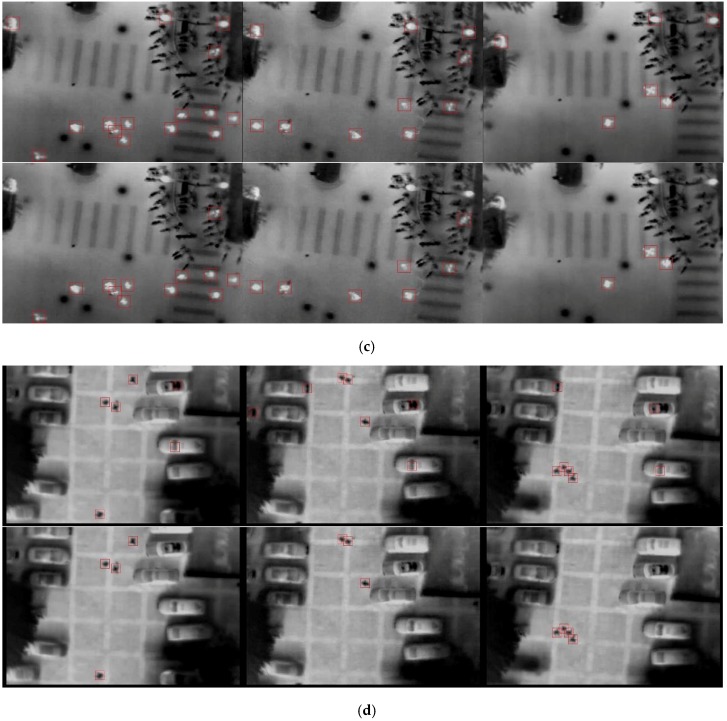
Pedestrian detection sample images for four scenes. (**a**) Pedestrian detection for Scene 2: the first row is the blob extraction results, and the second row is the classification results. (**b**) Pedestrian detection for Scene 3: the first row is the blob extraction results, and the second row is the classification results. (**c**) Pedestrian detection for Scene 4: the first row is the blob extraction results, and the second row is the classification results. (**d**) Pedestrian detection for Scene 4: the first row is the blob extraction results, and the second row is the classification results.

**Figure 15:**
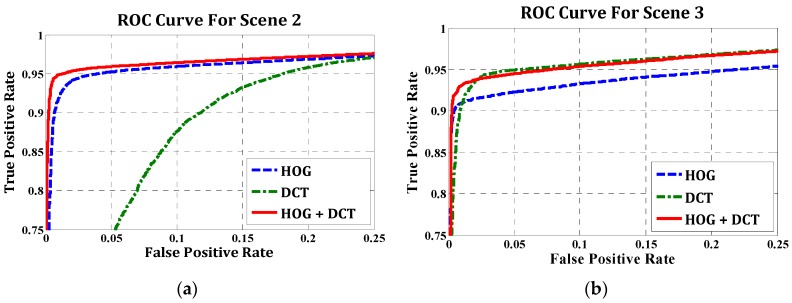

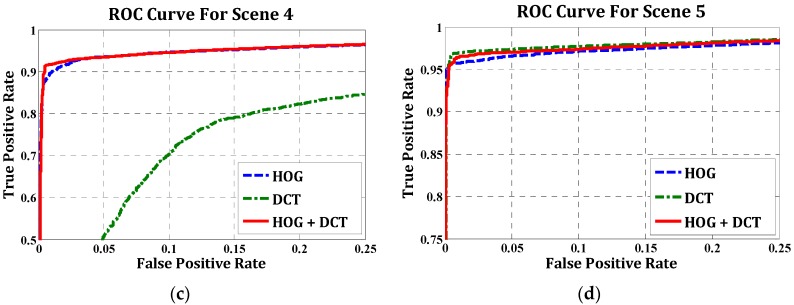
ROC curves for four training datasets. (**a**) scene 2, (**b**) scene 3 (**c**) scene 4 (**d**) scene 5.

In dataset “Scene 2”, pedestrians were easily distinguished from the background because of the high intensity (see Figure 14b); therefore, the training examples contained clear and detailed information about gradient and contour. Figure 15a illustrates the relative performance of three descriptors on this dataset. As shown, the HOG performed better than the DCT. This can be explained by the fact that the contour of pedestrian examples is clear and distinguishable between pedestrian and background examples from the salient local intensity gradient and edge direction information. The performance of the fusion descriptors is slightly better than HOG. Figure 14b shows the comparison of blob extraction results and corresponding blob classification results using the hybrid descriptor. Most wrong blobs were eliminated through classification. The combination of HOG and DCT strengthens the adaptability of feature descriptors, because the DCT descriptor can handle slight appearance changes and the halo effect. Meanwhile, the combination of both features increases the dimensionality of the feature vector, therefore containing more information for classification.

In dataset “Scene 3”, pedestrians were blurry due to the higher flight altitude than in the “Scene 2” dataset. As shown in Figure 15b, all three descriptors had good performance. With a false positive rate of 5%, more than 90% of pedestrians were classified correctly. As seen in the ROC curves, both the DCT and the fusion descriptors perform well and outperform the HOG. The possible reason is that the DCT descriptor extracts the key information from the example picture (top-left DCT coefficient); therefore, it has high discernibility for the blur appearance and imperceptible changes. The “Scene 4” dataset was captured with lower flight altitude than other datasets. Hence, the pedestrian blobs are larger and clearer, resulting in detailed gradient and edge information. However, this dataset is challenging and complex because of the interference of the road lamps and the vehicle headlights. As shown in Figure 14d (the top row), distinguishing the pedestrians and lamps is difficult. As we predicted, the DCT could not compete with the HOG descriptor due to the high similarity of true and false positive examples. As shown in Figure 15c, the true positive rate of DCT only roughly reaches half of the HOG and fusion descriptors. The fusion descriptor slightly outperformed the HOG, which was similar to “Scene 2”.

The last dataset “Scene 5” includes few pedestrian objects, which are shown as black blobs because of the high environment temperature. Figure 15d indicates the high classification precision of three descriptors, because fewer non-pedestrian candidate regions were detected in the blob extraction stage. Hence, the performance of the classifier is good.

As a result, the hybrid descriptor makes full use of the advantages of both HOG and DCT in representing the pedestrian feature, promotes the performance of the standalone use of HOG or DCT and tends to the better one. As shown in Table 2, the proposed detection method achieves a high precision in all datasets above 90% and a high recall rate of 84.84%–92.28%. The overall performance is encouraging. In practice, owing to the high similarity between pedestrian and cyclist blobs in aerial thermal top views, we regard cyclists as true positives. The used hyper-parameters in our experiments are presented in Table 4. Note that the range could be determined by ([k/2]−5, [k/2]+2).

### 4.2. Evaluation for Pedestrian Tracking

The performance of pedestrian tracking is evaluated by the Multiple Object Tracking Accuracy (MOTA) [34], as defined:

(11) $$\mathrm{MOTA}=1-\frac{\sum_{t}\left(m_{t}+FP_{t}+mme_{t}\right)}{\sum_{t}g_{t}}$$

where $m_{t}$, $FP_{t}$ and $mme_{t}$ are the number of missed pedestrians, of false positives and of mismatches, respectively, for time *t*, and $g_{t}$ is the number of actual total pedestrians. Note that in order to apply the proposed pedestrian tracking method, images have to be registered first. However, due to the drastic shaking of videos captured by the drone without the gimbal, few video clips can be registered well. In our test, only three available registered sequences from different scenes were selected. The basic information of test sequences is presented in Table 5. The m, FP, mme and g were counted on every image from the three test sequences. The obtained results are summarized in Table 6. For comparison, the tracking results from the original KLT tracker are also presented in Table 6.

As shown in Table 6, the overall performance of the proposed method is impressive with an average MOTA of 0.88; certainly, there is a room for improvement. Furthermore, compared to the KLT tracker, the proposed method with a secondary detection in search regions ensures continuous tracking and consistent trajectories. Therefore, the performance of the proposed method is significantly better than the original KLT tracker (more than 20% improvement, as shown in Table 6).

**Figure 16:**
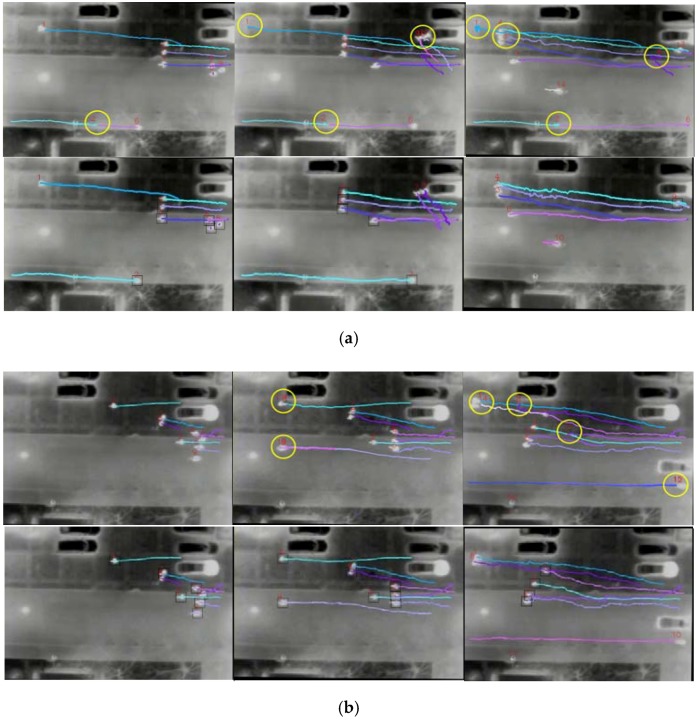

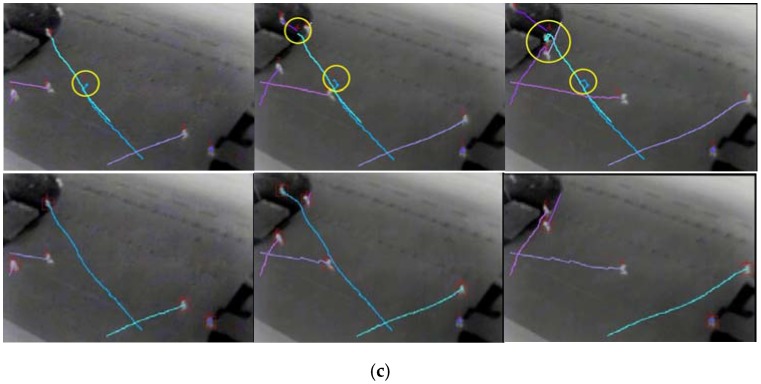
Pedestrian tracking sample images for three sequences. (**a**) Pedestrian tracking for Sequence 1: the first row is the KLT tracking results, and the second row is the proposed method tracking results; the yellow circles highlight the tracking errors. (**b**) Pedestrian tracking for Sequence 2: the first row is the KLT tracking results, and second row is the proposed method tracking results; the yellow circles highlight the tracking errors. (**c**) Pedestrian tracking for Sequence 3: the first row is the KLT tracking results, and the second row is the proposed method tracking results; the yellow circles highlight the tracking errors.

A possible reason for the different performances between the original KLT tracker and the proposed method is that the original KLT tracker generates more tracking misses (*i.e.*, $m_{t}$) and mismatches (*i.e.*, $mme_{t}$) due to the point drift (see Table 6). Tracking misses typically occur when pedestrians fail to be detected in the tracker initialization or secondary detection. This could be due to the low contrast or pedestrians walking too close. As shown in Figure 16a, second row, the pedestrian with ID 5 was missed when three pedestrians were walking too close, and the track point drifted away from this pedestrian. This created two tracking points for the pedestrian with ID 4. Because the tracker found the wrong object, a false positive occurred in subsequent frames. On the other hand, mismatch typically occurs due to the change of the tracking ID from previous frames, also called “ID switch”. In our three test sequence, mismatches are much less than tracking misses and false positives. This is partly because that the intersections between pedestrians’ walking paths could be more easily avoided from the vertical top view of the aerial images and partly due to the non-congested scenes tested in this research. Figure 16 demonstrates some examples of tracking errors, which are highlighted by yellow circles. Note here that the tracking errors include the tracking misses, false tracking and mismatches.

## 5. Pedestrian Tracking and Velocity Estimation

**Figure 17:**
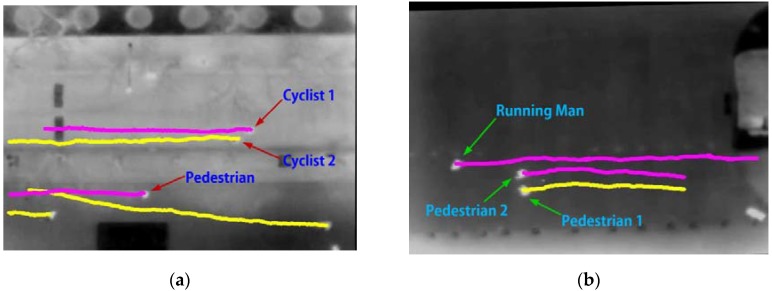
Cyclists and pedestrians trajectories. (**a**) Scene 1 and (**b**) Scene 2.

From the tracking results, the trajectories of pedestrians can be derived. Figure 17 presents some examples of pedestrian trajectories. However, from Figure 17, it is difficult to distinguish cyclists (or running men) from pedestrians, since from the top-view aerial thermal images, pedestrians and cyclists appear the same. This issue can be addressed by estimating the velocities of pedestrians and cyclists.

**Figure 18:**
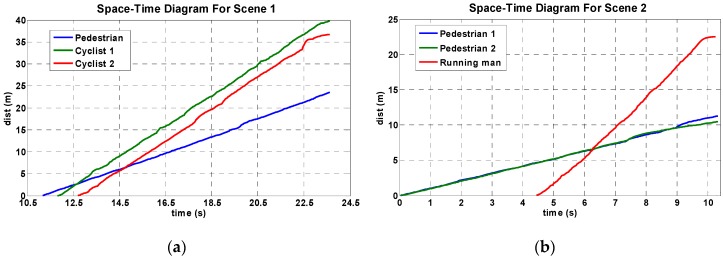
Pedestrian trajectory: space-time diagram. (**a**) Scene 1 and (**b**) Scene 2.

To estimate velocities, accurate trajectory data will be needed. Considering that the drift of track points may result in estimation errors of the true object center, the Kalman filter is used to process raw trajectory data. The Kalman filter computes the best estimate of the state vector (*i.e.*, location coordinates) in a way that minimizes the mean of the squared error according to the estimation of the past state and the measurement of the present state. Figure 18 presents the processed trajectory data, *i.e.*, time-space diagrams, of some detected objects. Since these time-space diagrams are close to straight lines, the velocity can be easily estimated by calculating the slopes of these lines. These diagrams can be used to distinguish cyclists (or running men) and walking pedestrians simply because the average velocity of cyclists (or running men) is larger than the velocity of walking pedestrians. The instantaneous velocity of an object can be estimated by the following equation:

(12) $$velocity\left(m/s\right)=\frac{X\left(pixels\right)\cdot Scale\left(m/pixel\right)\cdot FPS\left(frames/s\right)}{N\left(frames\right)}$$

where *X* is the motion distance of pedestrians; *Scale* represents the actual length of the unit pixel; *FPS* represents frames per second; and *N* is the total frames of pedestrian motion.

**Figure 19:**
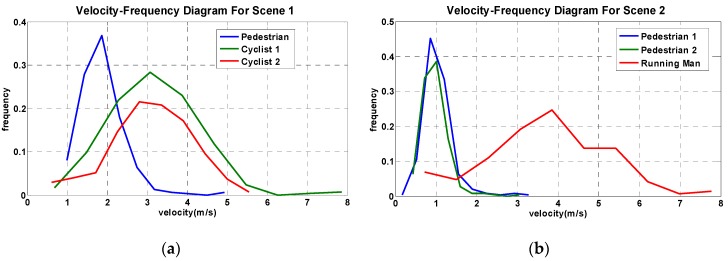
The frequency curve of the instantaneous velocity. (**a**) Scene 1 and (**b**) Scene 2.

With estimated instantaneous velocity data, the frequency curves of detected objects can be derived as shown in Figure 19. These frequency curves of the instantaneous velocity can also be used to distinguish pedestrians and cyclists (or running men). As shown in Figure 19a, the maximum frequency velocity of cyclists is approximately 3 m/s, which is significantly larger than the maximum frequency velocity of pedestrian of about 2 m/s. In addition, the instantaneous velocity of cyclists ranges roughly from 1 m/s–8 m/s, but for walking pedestrians, its instantaneous velocity range is more concentrated, because a severe offset occurred for tracking fast moving targets. Note, this method cannot be used to differentiate running men and cyclists, as running men and cyclists have very similar velocity profiles.

## 6. Discussion

The proposed blob extraction and blob classification scheme shows good detection results, as shown in Table 2. However, the accuracy of final pedestrian detection is impacted by two important factors: (1) different training sets during blob classification; and (2) the accuracy of blob extraction.

**Figure 20:**
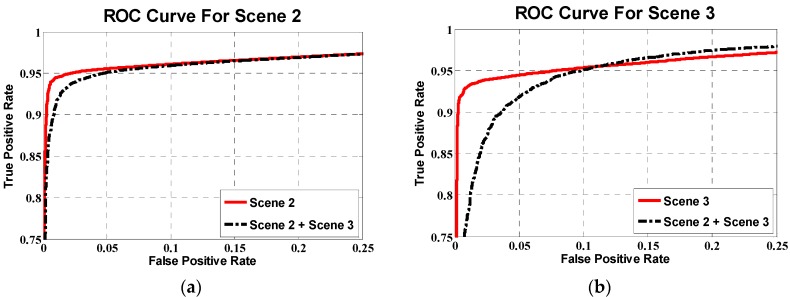
ROC curves for (**a**) Scene 2, (**b**) Scene 3.

Using Scenes 2 and 3 as an example, as shown in Table 1, the basic environment information (*i.e.*, temperature, flying height, *etc.*) of Scenes 2 and 3 is very similar, but the detection results are different. As shown in Table 2, the differences regarding precision and recall for Scenes 2 and 3 are 4.11% and 3.11%, respectively. Two reasons contribute to the difference of the final pedestrian detection:

(1) Different training sets during blob classifications. For different scenes, even if the environment is very similar, different training sets are used during the detection; because even a slight change of environment, such as temperature or flying height, will impact the positive and negative training samples. Therefore, one possible reason for the different detection results between Scenes 2 and 3 is because they use different training sets during blob classifications. To confirm this point, we merged the training blobs of Scenes 2 and 3 into an integrated dataset called “Scene 2 + Scene 3”. We train a new model with the “Scene 2 + Scene 3” dataset and test the classification performance in the Scenes 2 and 3 dataset, respectively. As shown in Figure 20, with new training sets, the detection results show differences.

(2) Accuracy of blob extraction. However, Figure 20 only shows that the new model is slightly worse than the original model. Further, referring to Figure 15a,b, the ROC curves show that the HOG + DCT classification model of Scene 2 is better than that of Scene 3, but the final detection results of Scene 2 are worse than those of Scene 3. Therefore, the training set may not be the main, at least not the only, factor. This leads to another important factor: Blob extraction.

To confirm our thought, we calculated the precision and recall of blob extraction in the Scene 2 and Scene 3 dataset. The following Table 7 presents the statistical results. As shown in Table 7, the precision and recall of Scene 3 are higher than those of Scene 2. This difference of blob extraction results is consistent with the difference of the final detection results. Thus, it is clear that one of the reasons (and maybe the main reason) why a detection difference does exist is due to the blob extraction difference.

Another indirect proof that blob extraction is one of the important reasons for the different detection results between Scenes 2 and 3 is that the Scene 3 dataset has less interference (such as lamps, vehicle headlights, *etc.*) than the Scene 2 dataset, therefore leading to the more accurate pedestrian candidate region extraction in Scene 3. Thus, there are less false pedestrian candidate regions in Scene 3, which results in higher precision and recall.

## 7. Conclusions

This paper proposes an approach to detect and track pedestrians in low-resolution aerial thermal infrared images. The proposed pedestrian detection method consists of two stages: blob extraction and blob classification. A blob extraction method based on regional gradient feature and geometric constraints filtering is first developed. This method can detect both bright and dark pedestrian objects in either daylight or at night. Furthermore, this method can work either with a static background or a moving platform. The proposed blob classification is based on a linear SVM, with a hybrid descriptor, which sophisticatedly combines the HOG and DCT descriptors. Experimental results showed the encouraging performance of the overall pedestrian detection method.

This paper further develops an effective method to track pedestrians and to derive pedestrian trajectories. The proposed method employs the feature tracker with the update of the detected pedestrian locations to accurately track pedestrian objects from the registered videos and to extract the motion trajectory data. The velocity characteristic of pedestrians and cyclists is also analyzed on the basis of velocity estimation.

**Figure 21:**
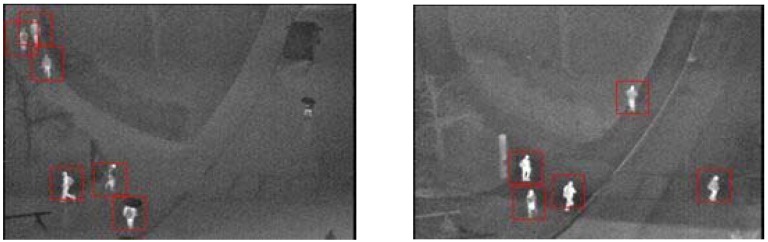
Blob extraction results in Ohio State University (OSU) thermal datasets.

We would like to point out that all methods described in this paper, individually, have been applied by other research. Some techniques, such as HOG and DCT features, SVM, video registration and KLT feature-based tracking, have been widely applied in much image processing research. We do not intend to claim the originality of these methods. In fact, the major contribution of this paper lies in how to apply these methods, as a whole, for pedestrian detection and tracking, particularly using thermal infrared images recorded from UAVs; such a topic, due to the low-resolution of imagery, platform motion, image instability and the relatively small size of the objects, has been considered as an extremely difficult challenge. The proposed comprehensive pedestrian detection and tracking system has successfully addressed this issue by nicely combining some existing classical detection and tracking methods with some relatively novel improvements, such as extracting pedestrian ROIs using a regional gradient feature and combining SVM with a hybrid HOG and DCT feature descriptor, which we have not seen in any direct applications in previous research based on our limited knowledge. In fact, the proposed blob extraction method can not only be used to extract pedestrian candidate regions in aerial infrared images, but also be used in the infrared images captured by roadside surveillance cameras mounted on low-altitude light poles with oblique views. As shown in Figure 21, using the thermal images provided by the Ohio State University (OSU) thermal public infrared dataset, our proposed blob extraction method can accurately extract pedestrian candidate regions.

More importantly, this research is expected to benefit many transportation applications, such as pedestrian-vehicle crash analysis, pedestrian facilities planning and traffic signal control (similar to [35]), Advanced Driver-Assistance System (ADAS) development, the multimodal performance measure [36] and pedestrian behavior study. Note that clearly the proposed pedestrian detection and tracking method can be further improved. For example, the current pedestrian detection method has difficulties dealing with the situations in which pedestrians are too close to each other, leading to the merging of pedestrian blobs caused by the halo effect. This problem brings significant missed detections. Furthermore, the employed video registration method works poorly when video pictures have drastic shaking due to wind or drone motion. Future work for addressing this issue will be considering mounting the camera gimbal on the drone to increase the stability of video pictures. More importantly, as a common drawback of all methods for object detection using thermal images, the low contrast of targets and background, especially in daylight, seriously affects the accurate detection. This issue cannot be addressed using our current method, but could be addressed by considering fusing thermal and visual images [15]. This will be a major future research topic.

## Figures and Tables

**Table 1:** Basic information of datasets.

Dataset	Resolution	Flying Altitude	Scenario	Date/Time	Temperature
**Scene 1**	720 × 480	40–70 m	Multi-scene	April/Afternoon, Night	10 °C–15 °C
**Scene 2**	720 × 480	50 m	Road	20 January/20:22 p.m.	5 °C
**Scene 3**	720 × 480	60 m	Road	19 January/19:46 p.m.	6 °C
**Scene 4**	720 × 480	40 m	Road	8 April/23:12 p.m.	14 °C
**Scene 5**	720 × 480	50 m	Road	8 July/16:12 p.m.	28 °C

**Table 2:** Detection statistical results.

Dataset	# Pedestrian	# TP	# FP	# FN	Precision	Recall
**Scene 1**	1320	1228	78	92	94.03%	93.03%
**Scene 2**	9214	8216	686	998	92.30%	89.17%
**Scene 3**	10,029	9255	344	774	96.41%	92.28%
**Scene 4**	5094	4322	396	772	91.61%	84.84%
**Scene 5**	3175	2923	197	252	93.69%	92.06%

**Table 3:** The number of training and test images for datasets.

Dataset	# Images	# Training	# Test	Training Blobs	Test Blobs
				# Positives/Negatives	# Positives/Negatives
**Scene 1**	No Training				
**Scene 2**	2783	544	2239	2347/9802	8339/35,215
**Scene 3**	2817	512	2305	2098/938	9237/4097
**Scene 4**	1446	365	1081	1560/881	4473/2817
**Scene 5**	1270	340	930	734/966	2984/4572

**Table 4:** Hyper-parameters used in the experiments.

Dataset	$T_{1}$	$T_{2}$	*k*	*Range*	α	*β*
**Scene 2**	3.5	3	29	(9, 16)	10	3
**Scene 3**	4	2	25	(7, 14)	10	3
**Scene 4**	3	2	35	(12, 19)	10	3
**Scene 5**	5	2	27	(8, 15)	10	3

**Table 5:** Basic information of sequences.

Dataset	Resolution	Flying Altitude	Scenario	Date/Time	Temperature
**Sequence 1**	720 × 480	50 m	Road	20 January/20:22 p.m.	5 °C
**Sequence 2**	720 × 480	50 m	Road	20 January/20:32 p.m.	5 °C
**Sequence 3**	720 × 480	45 m	Plaza	13 April/16:12 p.m.	18 °C

**Table 6:** Tracking statistical results.

Sequence	# Frames	#g	#m	#FP	#mme	MOTA
**Sequence 1**	635	3404	374	193	0	0.8334
**Sequence 1 KLT**	635	3404	379	956	5	0.6063
**Sequence 2**	992	4957	161	597	0	0.8471
**Sequence 2 KLT**	992	4957	198	1637	4	0.6290
**Sequence 3**	1186	3699	318	0	0	0.9140
**Sequence 3 KLT**	1186	3699	104	1367	5	0.6010

**Table 7:** Blob extraction statistical results.

Dataset	# Pedestrian	# TP	# FP	# FN	Precision	Recall
**Scene 2**	9214	8545	29,684	669	22.35%	92.74%
**Scene 3**	10,029	9633	3187	396	75.14%	96.05%

## Abbreviations

The following abbreviations are used in this manuscript:

UAVs: Unmanned Aerial Vehicles
SVM: Support Vector Machine
HOG: Histogram of Oriented Gradient
DCT: Discrete Cosine Transform
ROI: Region of Interest
CSM: Contour Saliency Map
